# Detecting poliovirus sequences in the sequence read archive database using bioinformatics tools

**DOI:** 10.3389/fbinf.2026.1856011

**Published:** 2026-08-12

**Authors:** Katie Farrell, Kevin Tang, Melchizedek Mashiku, Dawit Abay, Joseph Longo, Edward Ramos, Gabriel Leventhal-Douglas, Margaret Rohrbaugh, Paul Chenoweth, Cara C. Burns, Kun Zhao

**Affiliations:** 1 Contracting Agency to the Division of Viral Diseases, Centers for Disease Control and Prevention, Tanaq Management Services LLC, Chamblee, GA, United States; 2 Division of Core Laboratory Services and Response, Office of Laboratory Systems and Response, Centers for Disease Control & Prevention, Atlanta, GA, United States; 3 Contracting Agency to Centers for Disease Control and Prevention, General Dynamics Information Technology Inc., Atlanta, GA, United States; 4 Contracting Agency to Centers for Disease Control and Prevention, Pioneer Corporate Services Inc., Atlanta, GA, United States; 5 Division of Viral Diseases, National Center for Immunization and Respiratory Diseases, Centers for Disease Control and Prevention, Atlanta, GA, United States

**Keywords:** blast, Bowtie2, containment, poliovirus, sequence read archive, surveillance

## Abstract

One approach to find possible poliovirus sources that have not been detected by the Global Polio Eradication Initiative’s (GPEI) surveillance systems is to scan reads in the Sequence Read Archive (SRA) for potential poliovirus sequences. In the post-eradication era, the identification of poliovirus sequences in the SRA database could signal a potential biosafety risk which may set back the enormous achievements of the GPEI. To advance the use of the SRA database for detecting poliovirus, we hypothesized that bioinformatics alignment tools like Bowtie2, BLASTn, Magic-BLAST, MegaBLAST, STAT, and ElasticBLAST could distinguish between non-poliovirus and poliovirus reads from samples represented in the SRA database. Short poliovirus sequencing reads were simulated using poliovirus Sabin strain genomes. Simulation was also done for sequences other than poliovirus (referred here as “non-poliovirus reads”). Simulated reads were aligned to reference poliovirus genomes using different alignment tools to benchmark the accuracy and computing time of each tool. Parameters were also established to identify previously unknown poliovirus reads using percent identity and alignment length from BLASTn results. Bowtie2 was the most accurate and efficient tool, correctly identifying all simulated poliovirus reads. The STAT tool detected 99.6% of known control poliovirus accessions using the enterovirus query but only 77.6% using the poliovirus query, demonstrating strong but incomplete detection capability. This study demonstrates the feasibility of screening the SRA for poliovirus sequences as a tool to strengthen poliovirus containment and mitigate post-eradication risks, which can serve as an additional safety net for the GPEI.

## 1 Introduction

Since the launch of the Global Polio Eradication Initiative (GPEI) in 1988, wild poliovirus (WPV) cases have decreased by approximately 99.9%, with WPV type 2 officially eradicated in 2015 and type 3 eradicated in 2019 (Global Polio Eradication Initiative, 2026; Global Polio Eradication Initiative, 2024). WPV type 1 has continued to circulate in Pakistan and Afghanistan, with 99 cases in 2024, 52 in 2025, and 10 in 2026 of 8 July 2026 (Global Polio Eradication Initiative, 2026b). There are two types of polio vaccines, live attenuated oral poliovirus vaccine (OPV) and inactivated poliovirus vaccine (IPV) (World Health Organization, 2024). In rare cases, OPV can revert to regain neurovirulence and transmission properties that are equivalent to those of wild-type polioviruses. Mutated poliovirus strains can result in vaccine-associated paralytic poliomyelitis (VAPP) cases and seed vaccine-derived poliovirus (VDPV) outbreaks if allowed to circulate in under- or unimmunized populations. VDPV and VAPP are currently occurring in communities using OPV vaccines (World Health Organization, 2024; Pliaka et al., 2014). In 2024, there were 463 reported circulating vaccine-derived poliovirus (cVDPV) cases, 246 in 2025, and 80 in 2026 as of 8 July 2026 (Global Polio Eradication Initiative, 2026a).

Poliovirus consists of three serotypes, types 1, 2, and 3, that can all cause paralytic poliomyelitis or acute flaccid paralysis (AFP) in less than 1% of infections. Most infections are asymptomatic, but some individuals may experience mild symptoms (World Health Organization, 2024). Individuals excrete poliovirus in their feces for several weeks after infection, therefore the virus can be detected in sewage through environmental surveillance (Pliaka et al., 2014). Clinical AFP surveillance and environmental surveillance (ES) systems are critical for detecting poliovirus outbreaks, monitoring disease trends, and supporting eradication efforts. Both systems complement one another, as each has strengths that offset the other’s limitations. Clinical AFP surveillance is neither sensitive nor specific because most poliovirus infections go undetected; fewer than 1% of infections result in AFP, and AFP can arise from non-polio causes (World Health Organization, 2024). In a post-OPV, post eradication world, IPV will be the primary vaccine, and while it effectively prevents AFP, it induces limited intestinal immunity, allowing transmission to continue without clinical symptoms (World Health Organization, 2024). ES helps address these gaps, though it cannot be scaled globally due to technical and financial constraints (World Health Organization, 2023).

To strengthen poliovirus surveillance, we propose a new complementary approach that utilizes the Sequence Read Archive (SRA) to screen polio reads. It has the potential to provide global coverage, is not dependent on clinical case detection, and focuses directly on viral sequences. Because of the retrospective nature of the data, it will not provide real-time situational awareness. As a result, this approach is more suited to the post-eradication era, when the emphasis shifts from rapidly identifying and responding to outbreaks toward ensuring poliovirus is contained and monitoring for any breaches in containment.

The SRA database maintained by the National Center for Biotechnology Information (NCBI, NLM, NIH) serves as a data source for virus identification tools, such as Serratus (Sequence Read Archive, 2007; Edgar et al., 2022), which screens publicly available sequencing datasets. With the implementation of highly efficient alignment tools, the SRA database has the potential to identify emerging pathogens or incidental sequences on a global scale, offering a groundbreaking and novel approach to pathogen surveillance (Edgar et al., 2022). This study primarily focuses on datasets generated using short-read sequencing technologies because of their high accuracy, cost-effectiveness, and high-throughput data generation capabilities, as well as their widespread use in public sequencing repositories such as the SRA database where the vast majority are short reads (Ohta et al., 2017).

To evaluate the feasibility of using SRA data to support the GPEI, and to complement global environmental and AFP surveillance with a computational component, efficient and accurate bioinformatics alignment tools are needed. We hypothesize that bioinformatics alignment tools (Bowtie2 version 2.3.5.1 (Langmead and Salzberg, 2012), Magic-BLAST version 1.3.0 (Boratyn et al., 2019), BLASTn version 2.9.0 (Madden, 2013), MegaBLAST version 2.9.0 (Madden, 2013), ElasticBLAST 1.1.0 (Camacho et al., 2023), and the SRA Taxonomy Analysis Tool (STAT) (Katz et al., 2021)), could be used to enhance detection of poliovirus sequences in public datasets beyond those identified through existing surveillance systems. These bioinformatic tools were selected because they are freely available and widely used (Lambert et al., 2018). We further hypothesize that while the underlying principles of using bioinformatics tools remain consistent, parameter selection plays a critical role in determining which tool is the most effective at identifying potential poliovirus sequences among petabytes of SRA data. This report describes progress on the use of the bioinformatic tools to screen the SRA database for potential undetected poliovirus sequences.

## 2 Methods

### 2.1 Testing optimal method parameters for alignment tools

Alignment tools Bowtie2 version 2.3.5.1, Magic-BLAST version 1.3.0, BLASTn version 2.9.0, MegaBLAST version 2.9.0, were tested for optimal thread count, or the number of cores running the alignment in parallel, to ensure the most efficient options for alignment. All tools used local alignment but varied in seed length, or the number of nucleotides used to find matches. Bowtie2 had a default seed length of 20 bases, Magic-BLAST had 16 bases, MegaBLAST had 28 bases, and BLASTn had the shortest seed length of 11 bases. Three randomly selected SRA accessions containing reads that align to poliovirus (SRR10390621, SRR11412263, and SRR11412266) were aligned to the human poliovirus type 1 Sabin strain (GenBank ID: AY184219.1) type 2 Sabin strain (GenBank ID: AY184220.1), and type 3 Sabin strain (GenBank ID: AY184221.1) (Rezapkin et al., 1994). The optimal thread count was tested for Bowtie2, MegaBLAST, and BLAST using 4, 8, and 12; for Magic-BLAST, 2, 6, and 10 threads were tested, following software requirements. All commands are available in the Supplementary Material. The reference file was prepared following the specified method for each tool for creating a reference/database. The tools were tested using a high-performance computation (HPC) cluster with four nodes for each tool. The HPC cluster contained 110GB RAM, 330GB local scratch disk, 12 Intel Xeon5660 cores @ 2.8Ghz and 2 NVIDIA Tesla M2050 GPUs with 3GB RAM. Each tool was tested three times and the average time in minutes for the different thread counts was measured.

### 2.2 Sequence identity comparison to determine true poliovirus reads

Parameters that could be used to identify poliovirus reads using alignment length and percent identity were established by aligning poliovirus reference genome segments (5′UTR, a segment containing VP1, VP2, VP3 and VP4 (referred to as the capsid region); 2A, 2B, 2C, 3A, 3B, 3C and 3D (referred to as 2A-3D); and 3′ UTR) to all reference sequences for non-poliovirus genomes (consisting of non-polio enteroviruses, non-entero viruses, and all other organisms excluding the poliovirus genomes, in the NCBI RefSeq database) using BLASTn (Goldfarb et al., 2025). The total number of non-poliovirus organisms available in the database at the time was over 47 million organisms, with over 34 million bacterial organisms (Supplementary Table S1.) Alignment results show total alignment hits and were not deduplicated. To visualize parameters that could be used to identify poliovirus reads, a trendline was added to the graph as a schematic drawing of an empirically derived boundary. This trendline is used for visualization and discussion purposes for the poliovirus capsid region, further work would need to be conducted for the trendline to be considered as a distinct threshold to identify poliovirus.

### 2.3 Simulated read creation

All non-poliovirus viral organisms from the NCBI RefSeq database, 14,813 in total, were combined. The single poliovirus entry (NC_002058) in RefSeq was excluded to focus on non-poliovirus organisms that align to poliovirus. The reference sequences were downloaded between 1/22/2024 and 2/1/2024. The three complete genomes of Sabin poliovirus serotypes 1, 2, and 3 (AY184219, AY184220, and AY184221) were added to a separate file containing only the poliovirus genomes. All non-poliovirus viral Refseq entries (negative control) and the isolated poliovirus genomes (positive control) were used to create simulated reads at 10x coverage for 100, 150, 200, and 250 base pairs (bp). Simulation tool ART-MountRainier-2016–06-05 was used to simulate single ended reads at 10x coverage using the Illumina sequencing system MiSeq v3 (Huang et al., 2012).

### 2.4 Simulated read alignment

The simulated reads were aligned to the reference file containing AY184219, AY184220, and AY184221, prepared following instructions for each alignment tool. Bowtie2 version 2.3.5.1, Magic-BLAST version 1.3.0, BLASTn version 2.9.0, MegaBLAST version 2.9.0, and ElasticBLAST version 1.1.0 were used to align the simulated reads using one thread count for ElasticBLAST, four thread counts for Bowtie2, BLASTn, and MegaBLAST, and 10 thread counts for Magic-BLAST. Magic-BLAST, BLASTn, MegaBLAST, and Bowtie2 were run on a HPC cluster using four nodes. ElasticBLAST was run on the Google Cloud Platform on gke-version 1.24 with one node. BLASTn and ElasticBLAST were conducted with -num_alignment one to limit the number of alignments displayed for each query sequence to accommodate for the short default seed length compared to the other tools and lower the number of repeated read alignments. Each alignment was repeated three times, and the average time was recorded in minutes. Alignment results show total alignments; these results were not deduplicated post alignment. True positives are defined as reads that originated from the poliovirus genomes and aligned to poliovirus. False positives are defined as reads that originated from the non-poliovirus genomes and aligned to poliovirus. False negatives are defined as reads that originated from the poliovirus genomes and did not align to poliovirus. True negatives are defined as reads that originated from the non-poliovirus genomes and did not align to poliovirus. The average F1 score for each alignment tool was calculated using these definitions (Table 1).

**TABLE 1:** Simulated read count of RefSeq viral non-poliovirus genomes and poliovirus genomes to the poliovirus reference with the average time of alignment for each tool.

Simulated reads	Read (bp)	Total read count	Mega BLAST Count	Bowtie2 count	Magic- BLAST Count	BLASTn count	Elastic BLAST count
Poliovirus genomes 1, 2, 3	100	2220	2656	2220	2220	3100	3100
	150	1470	1941	1470	1470	2406	2406
	200	1110	1563	1110	1110	2073	2073
	250	870	1350	870	870	1806	1806
RefSeq non-poliovirus	100	46,863,594	463	366	1469	27,398,907	27,398,907
	150	31,215,040	593	339	1530	25,928,163	25,928,163
	200	23,389,198	535	279	1498	24,428,056	24,428,056
	250	18,696,233	577	311	1515	23,126,900	23,126,900
Average alignment time (minutes)			36.88	2.40	2.40	64.47	27.19
Average F1 score			0.7200	0.8893	0.6352	0.0001	0.0001

Simulated reads of all viral RefSeq non-poliovirus genomes and poliovirus genomes at 10x coverage and lengths of 100, 150, 200, and 250 bp were aligned by BLASTn, Bowtie2; Magic-BLAST, MegaBLAST, and ElasticBLAST, using the complete genomes of poliovirus serotypes 1, 2, and 3 as a reference. The total number of non-deduplicated reads that alignment tools aligned to the poliovirus genome are shown. The average length of time in minutes to align the total reads to the poliovirus genomes for each tool is shown. The average F1 score for each alignment tool is also shown.

### 2.5 SRA taxonomy analysis tool (STAT) references and comparison

The STAT tool was tested to compare the accuracy of STAT as well as to determine the best query to find poliovirus reads in sequence data. The STAT queries were conducted in the Google Cloud Platform on 07/27/23, which allowed access to the STAT database that contained results from NCBI running STAT on all accessions submitted to the SRA database since 2007 (Katz et al., 2021). NCBI runs STAT on accessions once they are uploaded to the SRA database and the STAT results are stored. Specific taxonomic nodes were queried using the NCBI taxonomy id (TaxId) that relates to each node. Three different queries were conducted starting with the first, which only queried for the enterovirus genus (NCBI:TaxId12059). The second queried for the Enterovirus coxsackiepol species (NCBI:TaxId138950), and the third queried for poliovirus serotypes 1, 2, and 3 (NCBI:TaxId12080, NCBI:TaxId12083, & NCBI:TaxId12086). Enterovirus, enterovirus C, and poliovirus serotypes 1, 2, and three were all queried with a minimum read count of one; in total there were three queries conducted. The enterovirus reference sequences used in STAT at the time this query was conducted are shown in Supplementary Table S2.

At the time the queries were conducted, there were 2,271 accessions in the SRA database that had been previously identified as known poliovirus samples, submitted by the CDC or other laboratories conducting poliovirus research or clinical trials. The samples covered 29 studies submitted by 16 institutions. These accessions were treated as control poliovirus samples to test the accuracy of STAT queries. STAT queries are available in the Supplementary Material.

## 3 Results

### 3.1 Optimal method parameters for alignment tools

The optimal parameter conditions to run the alignment tools on the HPC were determined before utilizing them to find poliovirus reads. The optimal thread counts to align SRA accessions SRR10390621, SRR11412263, and SRR11412266 for Bowtie2, MegaBLAST, and BLASTn were four threads with average run times of 8.29, 155.27, and 275.55 min and 10 threads with an average run time of 12.13 min for Magic-BLAST (Supplementary Table S3). Using 12 threads on the HPC took the longest for Bowtie2, BLASTn, and MegaBLAST with an average of 8.9, 164.95, and 282.3 min (Supplementary Table S3). Magic-BLAST took the longest with four threads at an average of 30.15 min to align the 3 SRA accessions (Supplementary Table S3).

### 3.2 Determining true poliovirus reads

**FIGURE 1:**
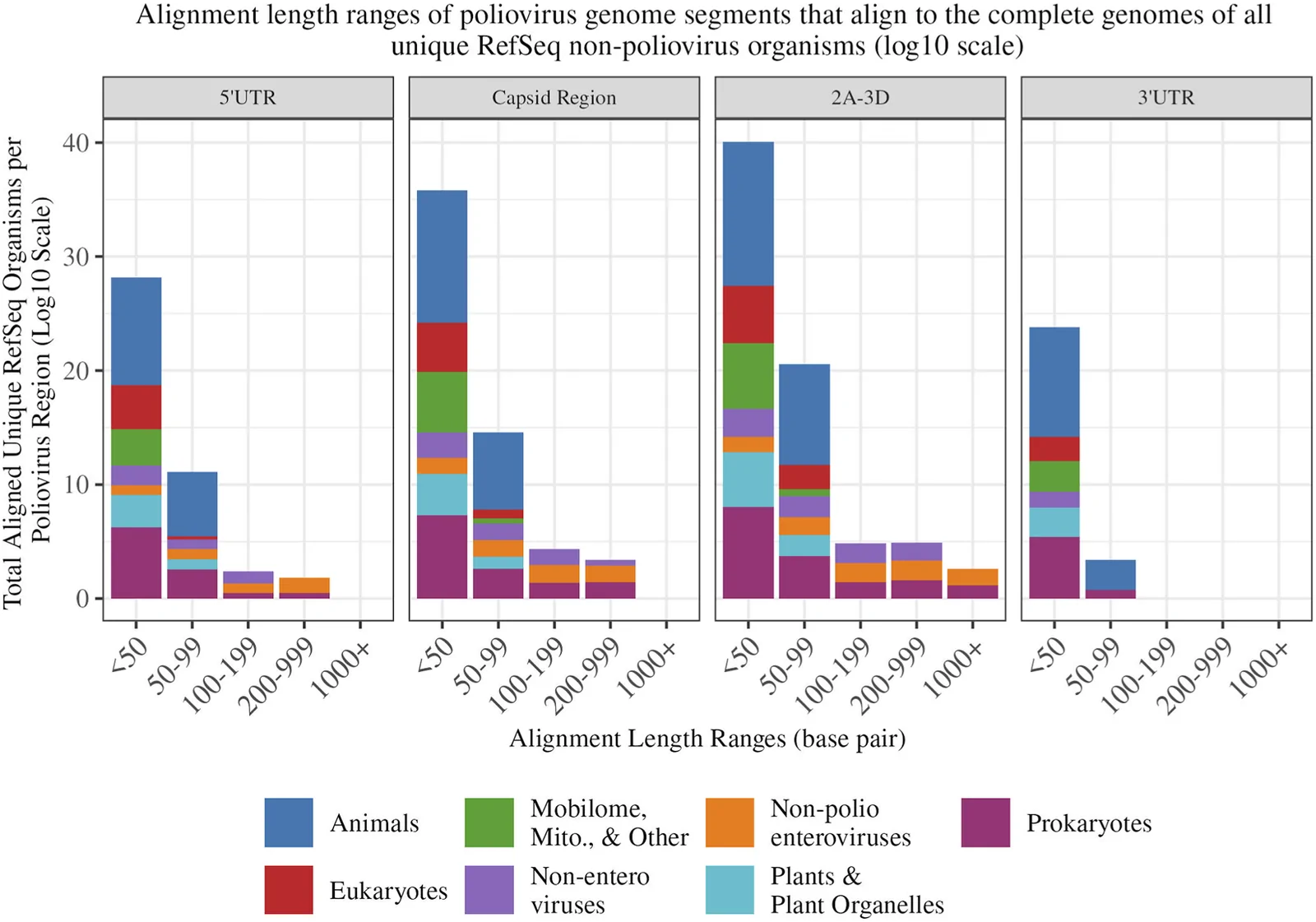
Graphs of poliovirus genome segments that align to the complete genomes of all non-poliovirus RefSeq organisms using BLASTn. Poliovirus genome segments were aligned with BLASTn against all non-poliovirus RefSeq organisms. The total non-deduplicated alignment hits per organisms that align with the poliovirus segments are shown. Non-poliovirus genomes represented by varying colors were grouped into the categories: 1) Animals (invertebrate, vertebrate mammalian, and vertebrate other), 2) Eukaryotes: (fungi and protozoa), 3) Mobilome, Mitochondria, and Other (plasmids, mitochondria, and other), 4) Non-enteroviruses, 5) Non-poliovirus Enteroviruses, 6) Plants and Plant Organelles (plants and plastids), and 7) Prokaryotes (archaea and bacteria). Genomes listed as “other” consist of unclassified metagenome samples and transposons. The y-axis represents the total number of complete genomes of all unique non-poliovirus RefSeq organisms (log10 scale) that align to the poliovirus genome segments from BLASTn alignment results.

Parameters needed to be established to create a potential threshold where poliovirus reads could easily be differentiated from non-poliovirus reads using alignment length and percent identity from BLASTn results. Poliovirus genome segments were aligned with BLASTn against all non-poliovirus RefSeq genomes. All unique non-poliovirus genomes distinguished by the alignment length ranges (bp) were plotted against poliovirus segments showing the total non-deduplicated RefSeq organism sequences that aligned to each poliovirus segment (Figure 1). The genome regions that formed alignments with the fewest non-poliovirus organism sequences were 3′UTR, VP1, VP4, and VP2. 2B, 3D and 3B had the most alignments of non-poliovirus organisms. Bacteria and vertebrates had the most sequences available in the RefSeq database and had the highest number of organisms align to poliovirus. However, mitochondria, viral, and sequences listed as “other” (consisting of unclassified metagenome samples and transposons) had the highest proportion of alignments made to the poliovirus segments out of the total available sequences per organism. 100% of all five genome sequences listed as “other”, 3.4% of all 17,330 available mitochondria sequences, 2.9% of all 18,719 viral sequences (excluding poliovirus), and 2% of all bacteria and vertebrate aligned to the poliovirus segments (Supplementary Table S1). Although “other”, mitochondria, and vertebrate sequences aligned proportionally higher, the longest alignment made for “other” sequences was 24 bp, whereas the longest alignment was 70 bp for mitochondria and 98 bp for vertebrate. Most of the alignments made between the non-poliovirus organisms and poliovirus segments were less than 50 bp (Figure 1). The longest alignments made for viral and bacterial sequences were 1398 bp, and 1347 bp, respectively. Poliovirus regions 2A-3D contained the longest alignments lengths with some alignments being over 1,000 bp. (Figure 1). However, poliovirus identification is generally not based on these regions because of the high recombination rates between poliovirus and other enteroviruses (Muslin et al., 2019). These long alignments indicated the possibility that the sequenced samples contained previously unidentified viral sequences in addition to bacterial sequences (i.e., cross-contamination, see supplement for additional details).

**FIGURE 2:**
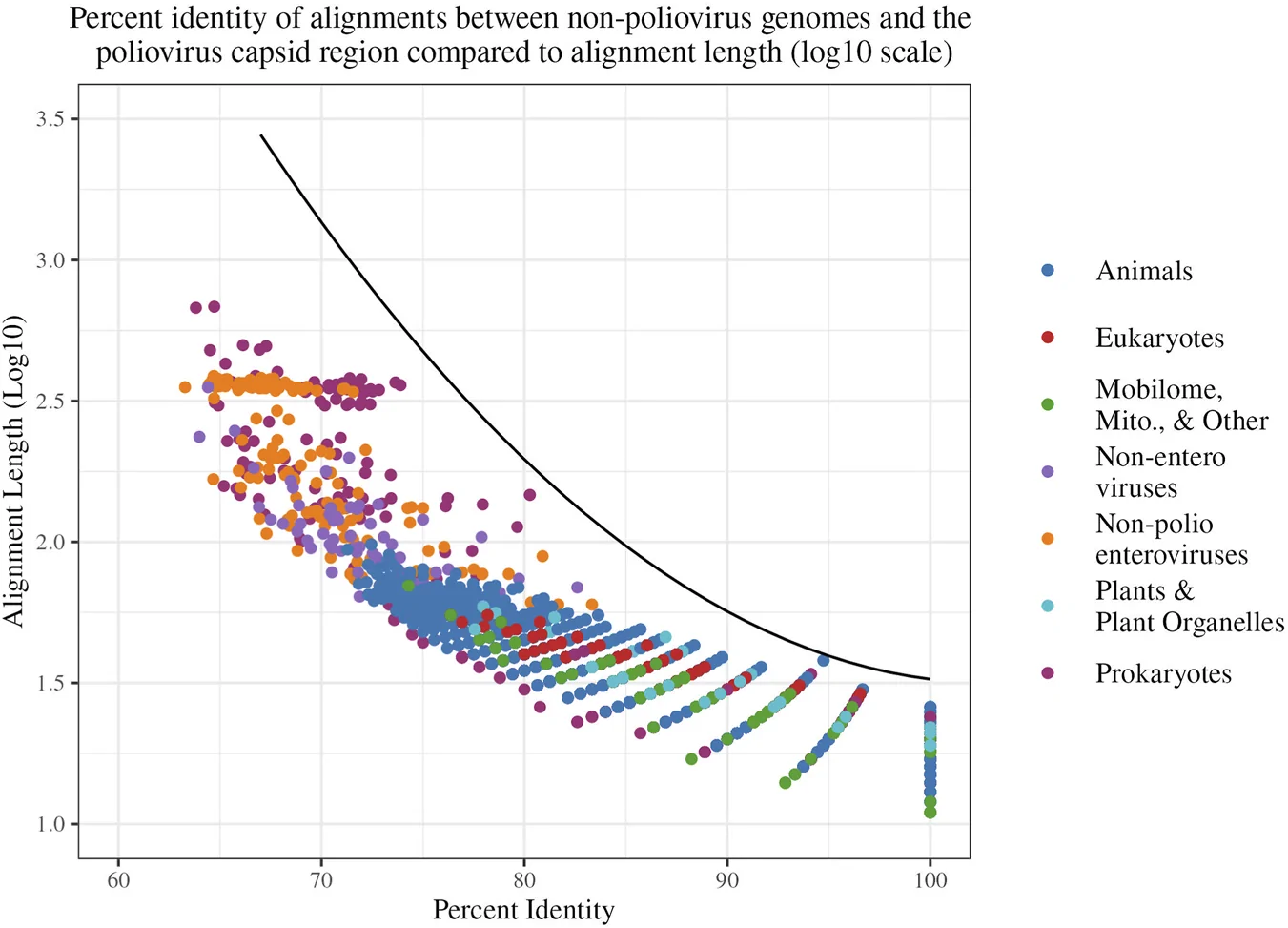
Scatterplot of alignment characteristics between poliovirus and various non-poliovirus reference organisms. Total non-deduplicated alignments made between the non-poliovirus genomes in the RefSeq database to the capsid region of poliovirus genome segments using BLASTn are shown. The sequence identity percentage after alignment between the non-poliovirus genome and the poliovirus segments were graphed with respect to the length of alignment. The x-axis represents the percent identity of each alignment hit. The y-axis represents the length of the alignment (log_10_ scale). Non-poliovirus genomes represented by varying colors were grouped into the categories: 1) Animals (invertebrate, vertebrate mammalian, and vertebrate other), 2) Eukaryotes: (fungi and protozoa), 3) Mobilome, Mitochondria, and Other (plasmids, mitochondria, and other), 4) Non-enteroviruses, 5) Non-poliovirus Enteroviruses, 6) Plants and Plant Organelles (plants and plastids), and 7) Prokaryotes (archaea and bacteria). Genomes listed as “other” consist of unclassified metagenome samples and transposons. The black trendline is a visual aid used to show the trend of non-poliovirus organisms aligned with the poliovirus genomes based on percent identity and alignment length. The line is a schematic drawing of an empirically derived boundary only to be used for visualization purposes for the poliovirus capsid region, not to be used as a distinct threshold to identify poliovirus.

All Sabin poliovirus genome segments were aligned with BLASTn against all non-poliovirus RefSeq genomes, and the corresponding sequence identity percentage for each alignment hit was plotted against the length of the alignment (sequence overlap) for all total non-deduplicated alignments made to the poliovirus capsid region (Figure 2). The length of the sequence overlaps in the capsid region was graphed as a Log10 scale to easily observe results as the range of sequence length was 11–1347 bp. An inverse relationship was observed between the alignment length and sequence identity between poliovirus and non-poliovirus genomes. That is, as the alignment length increases, the sequence percent identity decreases for non-poliovirus organisms. The maximal sequence identity (100%) was observed at a maximal alignment length of 27 bp, whereas the maximal alignment length (1347 bp) had only 69% in sequence identity. Non-poliovirus organisms shared sequence identity ranging from 63%–100% with alignment lengths ranging from 11–1398 bp.

All non-poliovirus RefSeq organisms that aligned to the capsid region of the poliovirus genomes are shown in Figure 2. Considerably fewer organisms aligned to the capsid region compared to more conserved regions of the poliovirus genome (Supplementary Table S1). The total number of unique RefSeq organisms that aligned with poliovirus is shown with the corresponding total number of alignment hits (Supplementary Table S1). Reads that align to the poliovirus capsid regions were considered poliovirus reads if the percent identity and alignment length met the threshold by being above the schematically drawn trendline (Figure 2).

### 3.3 Simulated reads vs. alignment tools

After establishing parameter conditions for the alignment tools and creating a threshold for poliovirus reads, BLASTn, ElasticBLAST, MegaBLAST, Magic-BLAST, and Bowtie2 were tested to determine the most accurate and efficient alignment tool by aligning simulated read files to the Sabin poliovirus genomes. The total number of simulated 10x reads created from the RefSeq sequences without any poliovirus entries for 100, 150, 200, and 250 bp was 46.9 million, 31.2 million, 23.4 million, and 18.7 million reads respectively. Since there were no poliovirus sequences included, there were 0 poliovirus reads. The isolated Sabin poliovirus serotypes 1, 2, and three simulated reads created from AY184219, AY184220, and AY184221 had 2220, 1470, 1110, and 870 total reads for 100, 150, 200, and 250 bp, respectively. Since only poliovirus reads were included in the sequence file, the total number of reads were the same as poliovirus reads.

BLASTn and ElasticBLAST aligned the most simulated non-poliovirus reads to the poliovirus reference sequences with over 20 million aligned reads (Table 1). Bowtie2 had the lowest number of aligned non-poliovirus reads to the poliovirus genome with 366, 339, 279, and 311 (Table 1). BLASTn and ElasticBLAST also aligned the most simulated poliovirus reads to the poliovirus reference sequences. BLASTn and ElasticBLAST aligned 3100, 2406, 2073, and 1806 reads, for 100, 150, 200, and 250 bp, respectively. These tools aligned more poliovirus reads than what was present in the simulated files likely due to very short reads or repeated alignments. Bowtie2 was the only alignment tool to find the exact number of possible poliovirus reads in the simulated read files while aligning a low number of non-poliovirus reads (Table 1) with the highest average F1 score of 0.89. Magic-BLAST also aligned all possible poliovirus reads in the simulated read files and aligned a low number of non-poliovirus reads but was not as accurate as Bowtie2 with an average F1 score of 0.64.

The average length of time BLASTn, Bowtie2, Magic-BLAST, MegaBLAST, and ElasticBLAST required to align the simulated reads was recorded in minutes (Table 1). Magic-BLAST and Bowtie2 aligned the simulated read files most quickly, with an average of 2.40 min. MegaBLAST aligned read files with an average of 36.88 min, ElasticBLAST with 27.19 min, and BLASTn took the longest with 64.47 min.

### 3.4 SRA taxonomy analysis tool (STAT) queries

Although STAT was not originally designed to predict or identify poliovirus, the tool was evaluated to determine if it can be utilized to narrow down the search for accessions with poliovirus reads instead of screening every accession in the SRA database. The STAT tool was used to query all accessions in the SRA database, to identify those accessions that contain at least one read mapped to poliovirus 1, 2, and 3, enterovirus C, and enterovirus. While querying poliovirus directly using STAT, a total of 5,557 accessions were identified as containing at least one read mapped to poliovirus genomes. Among the 2,271 SRA control poliovirus accessions, the poliovirus query detected 1,763. Because poliovirus belongs to the Enterovirus genus within the family Picornaviridae, the enterovirus C query and the enterovirus query were further tested to determine whether they could identify all 2,271 control accessions. Expanding the parameters to include all members of the Enterovirus genus improved STAT’s performance from 1,763 to 2,263 correctly identified control accessions. However, this improvement came at the cost of a fivefold increase in non-control identifications, from 3,794 to 178,736 accessions, and eight control samples remained undetected (Table 2). Out of the eight accessions, two were missing from the STAT database and could not be accessed by the STAT query, which is why they were not included in the query results at the time. This was determined via communication with NCBI. All available accessions that did not map to poliovirus were from studies that sequenced stool samples of recipients after receiving the OPV vaccine (Valesano et al., 2021; Tan et al., 2020).

**TABLE 2:** STAT query results showing control and non-control accessions identified by STAT.

STAT Tax ID	Total accessions identified by STAT query	Control accessions identified by STAT (matches to 2,271 SRA control poliovirus accessions)	Non-control accessions identified by STAT
Poliovirus serotypes 1, 2, 3	5,557	1,763	3,794
Enterovirus C	14,180	2,238	11,942
Enterovirus	180,999	2,263	178,736

The three different queries were conducted using STAT, to screen all accessions in the SRA, database since 2007. The STAT, TaxIds included enterovirus genus (NCBI:TaxId12059), enterovirus C species (NCBI:TaxId138950), and poliovirus serotypes 1, 2, and 3 (NCBI:TaxId12080, NCBI:TaxId12083, & NCBI:TaxId12086). The total number of accessions that had at least one read map to each TaxId after conducting the three queries is shown.

## 4 Discussion

Utilizing publicly available data in the SRA database to identify poliovirus sequences offers a valuable and distinct contribution to the GPEI. Unlike typical metagenomics or other bioinformatics activities that focus on broader pathogen detection or exploratory analysis, this approach is purposefully designed for a post-eradication landscape, where poliovirus is no longer circulating, and any remaining poliovirus present would pose a significant risk. In this context, containment requires casting the widest possible net with the smallest possible mesh size to ensure that no potential event is missed.

While the concept may seem straightforward, preliminary testing of available bioinformatic tools revealed that screening the SRA database poses challenges due to the large number of short reads from non-poliovirus organisms that can align to the poliovirus genome (Figures 1, 2). Our work also shows that the task is feasible with the use of an efficient and accurate alignment tool. Bowtie2 was identified as the most suitable tool for this purpose because it demonstrated the fastest performance, the highest accuracy in aligning true poliovirus reads, the lowest rate of false-positive alignments, and the highest average F1 score (Table 1).

A parameter set for differentiating between poliovirus and non-poliovirus reads was established based on alignment length and percent identity (Figure 2). Reads that fall above the trendline and that meet both alignment length and percent identity thresholds could be considered poliovirus, since no other organisms were found to share those parameter conditions. The capsid region was used for preliminary threshold development because, although the VP1 region remains the gold standard for poliovirus identification and molecular characterization (Colston and Racaniello, 1995; Oberste et al., 1999), compared with VP1 alone, the capsid region provides greater genomic coverage and additional sequence information. This can improve the resolution of molecular characterization and genotyping analyses (Global Polio Laboratory Network, 2024). Additionally, most alignments were between poliovirus segments and vertebrate and bacterial samples, likely due to the disproportionate representation of these organisms in the RefSeq database. Proportionally to the number of entries in the RefSeq, mitochondria, viral, and sequences listed as “other” aligned more frequently to the poliovirus genomes. These observations suggest that non-poliovirus reads could align to the poliovirus genome, especially when read lengths are short and sequence identity is low, as observed during alignment tool testing. This highlights the critical role of parameter settings used by bioinformatics tools in ensuring accurate identification.

Using the STAT tool to identify poliovirus reads in sequence data from the SRA database is possible and extremely efficient since the tool can screen all accessions in the SRA database within minutes (Katz et al., 2021). However, supporting GPEI efforts will require bioinformatics tools that minimize the risk of missing true poliovirus detections. When the detection threshold was relaxed to include a single read mapping to the Enterovirus genome, STAT achieved 99.6% relevant identification (2,263/2,271 matched to polio). However, this relaxation also produced a fivefold increase in non-control accessions identified by STAT, indicating reduced specificity and higher downstream validation workload (Table 2). This finding further underscores the necessity of using an efficient, poliovirus-specific alignment tool for downstream validation following initial screening with STAT. The increased non-control accessions found by STAT were not further evaluated for poliovirus identification and this study does not claim to identify undetected poliovirus positive SRA accessions.

Of the eight undetected control accessions, two were missing from the STAT database due to delayed updates, highlighting that the accuracy of STAT-based screening depends on how frequently the database is refreshed. The remaining six accessions, including SRR11968353 (33% VP1 identity), were below the 75% VP1 identity threshold for poliovirus (Oberste et al., 1999) and likely represent incomplete or degraded genomes, warranting re-sequencing or further sample processing optimization to confirm the presence of poliovirus.

It is also important to note that the reference sequences used by STAT for enterovirus, enterovirus C, and poliovirus serotypes 1, 2, and three differ from those in the RefSeq database and from those used in simulated read testing. Specifically, STAT uses the Sabin and Lansing strains as poliovirus references, rather than the Sabin strain for all three serotypes, as is standard in accredited laboratories supporting GPEI (Supplementary Table S2). Despite these limitations, STAT would be a useful tool to reduce the number of accessions that need to be aligned with Bowtie2. Instead of aligning millions of accessions in the SRA database, only accessions identified by STAT with reads that map to the viral family would be selected for alignment, provided the data is available in the STAT database. Addressing missing accessions is a key challenge for ensuring accurate surveillance, as their absence could mask true poliovirus detections. To minimize these uncertainties, multiple complementary tools should be used for SRA screening rather than relying on a single tool such as STAT alone. For example, further screening of STAT-identified accessions using Bowtie2 is recommended, as STAT results do not provide key metrics such as coverage, percent identity, alignment regions, or single-nucleotide polymorphisms (SNPs).

This study has limitations. First, this study was limited to short-read sequencing data, and further investigation is needed to determine the extent to which these observations apply to long-read sequencing data. Secondly, further investigation is needed to utilize the trendline as a distinct threshold. Thirdly, programmatically, while not intended to play a role in near real-time detection and response given the retrospective nature of the data, our proposal to contain poliovirus from sequences matched in the SRA database has not yet been tested in the real world. In summary, screening the SRA database for poliovirus reads for surveillance and containment purposes is bioinformatically feasible using Bowtie2, and STAT, with appropriate parameter thresholds such as alignment length and percent identity, which enable differentiation between non-poliovirus and poliovirus reads. Screening public sequence databases could be a key component in supporting poliovirus eradication and containment efforts in the post-eradication era.

## Data Availability

All commands, queries, and datasets for this study can be found in the PASS/DPS Repository https://github.com/CDCgov/PASS/tree/master/DPS.
