# Supplementary material for: Detecting poliovirus sequences in the sequence read archive database using bioinformatics tools
Source: Front Bioinform. 2026 Aug 12;6:1856011. doi: 10.3389/fbinf.2026.1856011 (PMC13506724; doi:10.3389/fbinf.2026.1856011)
Supplement: Supplementary file 1 [file Supplementaryfile1.docx]

Supplementary Material

**Commands**

**Testing Optimal Method Parameters for Alignment Tools:**

BLASTn

seqtk seq -A acc.fq | blastn -task blastn -db ~/references/blast/sabin/sabin -num_threads 4 -num_alignments 1 -outfmt "6 qseqid sseqid pident qlen length mismatch gapopen qstart qend sstart send evalue bitscore qseq sseq" >> acc.txt

seqtk seq -A acc.fq | blastn -task blastn -db ~/references/blast/sabin/sabin -num_threads 8 -num_alignments 1 -outfmt "6 qseqid sseqid pident qlen length mismatch gapopen qstart qend sstart send evalue bitscore qseq sseq" >> acc.txt

seqtk seq -A acc.fq | blastn -task blastn -db ~/references/blast/sabin/sabin -num_threads 12 -num_alignments 1 -outfmt "6 qseqid sseqid pident qlen length mismatch gapopen qstart qend sstart send evalue bitscore qseq sseq" >> acc.txt

MegaBLAST

seqtk seq -A acc.fq | blastn -task megablast -db ~/references/blast/sabin/sabin -num_threads 4 -outfmt "6 qseqid sseqid pident qlen length mismatch gapopen qstart qend sstart send evalue bitscore qseq sseq" >> acc.txt

seqtk seq -A acc.fq | blastn -task megablast -db ~/references/blast/sabin/sabin -num_threads 8 -outfmt "6 qseqid sseqid pident qlen length mismatch gapopen qstart qend sstart send evalue bitscore qseq sseq" >> acc.txt

seqtk seq -A acc.fq | blastn -task megablast -db ~/references/blast/sabin/sabin -num_threads 12 -outfmt "6 qseqid sseqid pident qlen length mismatch gapopen qstart qend sstart send evalue bitscore qseq sseq" >> acc.txt

Bowtie2

bowtie2 -p 4 --sensitive-local -N 1 -t -x ~/references/sabin/sabin -q acc.fq --un acc_unaligned.fq --al acc_aligned.fq -S acc.sam

bowtie2 -p 8 --sensitive-local -N 1 -t -x ~/references/sabin/sabin -q acc.fq --un acc_unaligned.fq --al acc_aligned.fq -S acc.sam

bowtie2 -p 12 --sensitive-local -N 1 -t -x ~/references/sabin/sabin -q acc.fq --un acc_unaligned.fq --al acc_aligned.fq -S acc.sam

Magic-BLAST

magicblast -query accession.fq -db ~/references/blast/sabin/sabin -num_threads 2 -outfmt tabular -infmt fastq > accession.txt

magicblast -query accession.fq -db ~/references/blast/sabin/sabin -num_threads 6-outfmt tabular -infmt fastq > accession.txt

magicblast -query accession.fq -db ~/references/blast/sabin/sabin -num_threads 10 -outfmt tabular -infmt fastq > accession.txt

**Sequence Identity Comparison to Determine True Poliovirus Reads:**

Org: archaea, bacteria, fungi, invertebrate, mitochondrion, non-enteroviruses, non-poliovirus enteroviruses, plants, plasmids, plastids, protozoa, vertebrate (mam), vertebrate (other), and other genomes

blastn -task blastn -query AY184219m.fasta -db refseq_org -outfmt "6 qseqid sseqid pident qlen length" >> org_1.txt

blastn -task blastn -query AY184220m.fasta -db refseq_org -outfmt "6 qseqid sseqid pident qlen length" >> org_2.txt

blastn -task blastn -query AY184221m.fasta -db refseq_org -outfmt "6 qseqid sseqid pident qlen length" >> org_3.txt

**Simulated read creation:**

#ART simulated read creation for RefSeq viral organisms without poliovirus entry: NC_002058

art_illumina -ss MSv3 -i refseq_wopolio.fasta -l 100 -f 10 -o refseq_wopolio100

art_illumina -ss MSv3 -i refseq_wopolio.fasta -l 150 -f 10 -o refseq_wopolio150

art_illumina -ss MSv3 -i refseq_wopolio.fasta -l 200 -f 10 -o refseq_wopolio200

#art_illumina -ss MSv3 -i refseq_wopolio.fasta -l 250 -f 10 -o refseq_wopolio250

##ART simulated read creation of Sabin poliovirus serotypes 1, 2, 3 reference

art_illumina -ss MSv3 -i sabin.fasta -l 100 -f 10 -o sabin100_10x

art_illumina -ss MSv3 -i sabin.fasta -l 150 -f 10 -o sabin150_10x

art_illumina -ss MSv3 -i sabin.fasta -l 200 -f 10 -o sabin200_10x

art_illumina -ss MSv3 -i sabin.fasta -l 250 -f 10 -o sabin250_10x

**Simulated Read Alignment**

Readlength: 100, 150, 200, 250

Type: refseq_wopolio, polio

bowtie2 -p 4 --sensitive-local -N 1 -t -x ~/references/sabin/sabin -q /simreads/readlength_type.fq --un /readlength_type_unaligned.fq --al readlength_type _bowtie2.fq -S readlength_type .sam

magicblast -query /simreads/readlength_type.fq -db ~/references/blast/sabin/sabin -num_threads 10 -outfmt tabular -infmt fastq > /simreads/readlength_type_magic.txt

seqtk seq -A /simreads/readlength_type.fq | blastn -task megablast -db ~/references/blast/sabin/sabin -num_threads 4 -outfmt "6 qseqid sseqid pident qlen length mismatch gapopen qstart qend sstart send evalue bitscore qseq sseq" >> readlength_type _mega.txt

seqtk seq -A /simreads/readlength_type.fq | blastn -task blastn -db ~/references/blast/sabin/sabin -num_threads 4 -num_alignments 1 -outfmt "6 qseqid sseqid pident qlen length mismatch gapopen qstart qend sstart send evalue bitscore qseq sseq" >> readlength_type _blastn.txt

[elasticblast]

program = blastn

db = gs://sabindb/sabindb

queries = gs://sim_fa/ readlength_type.fasta

results = gs://results/blast

options = -task blastn -num_alignments 1 -outfmt "6 qseqid sseqid pident qlen length mismatch gapopen qstart qend sstart send evalue bitscore qseq sseq"

**STAT Queries:**

**Tax_id=**

12059 (Enterovirus)

138950 (Enterovirus coxsackiepol)

12080, 12083, 12086 (poliovirus types 1, 2, and 3)

query = """

#standardSQL

WITH

  span AS (

  SELECT

    acc,

    ileft AS rileft,

    iright AS riright

  FROM

    nih-sra-datastore.sra_tax_analysis_tool.tax_analysis

  WHERE

    tax_id = 12059

    )

SELECT

  acc,

  name,

  total_count,

  self_count,

  center_name,

  librarysource,

  organism,

  releasedate,

  geo_loc_name_sam,

  attributes

FROM

  nih-sra-datastore.sra_tax_analysis_tool.tax_analysis

JOIN

  nih-sra-datastore.sra.metadata

USING

  (acc)

JOIN

  span

USING

  (acc)

WHERE

  (ileft>=rileft

    AND iright<=riright)

  AND (total_count>1

    OR organism='wastewater metagenome')

  AND CAST(releasedate AS date) > DATE_SUB(CURRENT_DATE(), INTERVAL 192 month)

ORDER BY

  releasedate DESC,

  acc,

  total_count DESC

"""

| **Organism** | **Total RefSeq organisms** | **RefSeq organism sequences that align to poliovirus** | **Proportion of organism sequences that aligned to poliovirus out of total available (%)** | **Total alignments to a segment of the full poliovirus genome** | **RefSeq organism sequences that aligned to the capsid region of poliovirus** | **Total alignments to the capsid region of poliovirus** |
| --- | --- | --- | --- | --- | --- | --- |
| **Archaea** | 109,840 | 364 | 0.33 | 419 | 111 | 133 |
| **Bacteria** | 34,401,040 | 639,513 | 1.86 | 859,407 | 164,306 | 187,909 |
| **Fungi** | 117,479 | 557 | 0.47 | 1,200 | 180 | 368 |
| **Invertebrate** | 2,671,000 | 510 | 0.02 | 665 | 189 | 214 |
| **Mitochondrion** | 17,330 | 588 | 3.39 | 787 | 289 | 320 |
| **Other** | 5 | 5 | 100 | 59 | 5 | 21 |
| **Plant** | 2,133,306 | 586 | 0.03 | 712 | 139 | 145 |
| **Plasmid** | 83,269 | 274 | 0.33 | 293 | 73 | 73 |
| **Plastid** | 14,116 | 274 | 1.94 | 304 | 37 | 40 |
| **Protozoa** | 170,662 | 607 | 0.36 | 711 | 127 | 141 |
| **Vertebrate (mam)** | 3,423,710 | 52,855 | 1.54 | 681,388 | 21,924 | 110,733 |
| **Vertebrate (other)** | 3,896,799 | 76,162 | 1.95 | 647,703 | 28,824 | 135,884 |
| **Virus** | 18,719 | 538 | 2.87 | 1,630 | 201 | 418 |
| **Total** | 47,057,275 | 772,833 | NA | 2,195,278 | 216,405 | 436,399 |

**Supplementary Table 1. Table with all organisms available in the RefSeq database including the unique organisms that poliovirus aligns with, the proportion of organism sequences that aligned to poliovirus (out of total available), and the total number of alignment hits after BLASTn.** All non-poliovirus RefSeq organisms available in the RefSeq database were used as references to align segmented poliovirus genomes 1, 2, and 3 using BLASTn. All organisms with reads aligned to the poliovirus genomes are shown, as well as the total number of alignment hits after using BLASTn.

|  | **STAT Reference Sequences** | |
| --- | --- | --- |
| **Name** | **GenBank ID** | **Poliovirus Strain** |
| Enterovirus | X96903.1  X96904.1  X96905.1  X96906.1  X96902.1  X96901.1  X96912.1  X96910.1  X96907.1  X96908.1  X96899.1  X96900.1  X96897.1  X96898.1  X96909.1  X96911.1  Y11010.1  Y11011.1  Y11012.1  Y11013.1  Y11014.1  Y11015.1  Y11016.1 |  |
|  |  |  |
|  |  |  |
|  |  |  |
|  |  |  |
|  |  |  |
|  |  |  |
|  |  |  |
| Enterovirus C | NC002058.3 | Mahoney |
| Poliovirus 1 | V01150.1 | Sabin |
| Poliovirus 2 | M12197.1 | Lansing |
| Poliovirus 3 | X00925.1 | Sabin |

**Supplementary Table 2**. **Table showing all GenBank IDs used as reference sequences by the SRA Taxonomy Analysis Tool for Enterovirus, Enterovirus C, and Poliovirus serotypes 1, 2, and 3.**

 A

| **Thread count** | **Bowtie2 (min)** | **BLASTn (min)** | **MegaBLAST (min)** |
| --- | --- | --- | --- |
| 4 | 8.29 | 275.55 | 155.27 |
| 8 | 8.78 | 280.47 | 167.24 |
| 12 | 8.9 | 282.3 | 164.95 |

B

| **Thread count** | **Magic-BLAST (min)** |
| --- | --- |
| 2 | 30.15 |
| 6 | 15.5 |
| 10 | 12.13 |

**Supplementary Table 3. Tables showing the average time to align 3 accessions using varying thread counts on the HPC for Bowtie2, BLASTn, MegaBLAST, and Magic-BLAST.** Reads of SRR10390621, SRR11412263, and SRR11412266 were aligned with Bowtie2, MegaBLAST, BLAST, and Magic-BLAST. Thread counts 4, 8, and 12 were tested for Bowtie2, MegaBLAST, BLAST **(A)** and 2, 6, and 10 for Magic-BLAST **(B)** on HPC. The length of time for each alignment was recorded in minutes.

*30 bacterial RefSeq sequence entries were removed from Figure 2 due to potential enterovirus contaminants. To investigate this possibility, the RefSeq entries in question were aligned against all available nucleotide (nt) databases from NCBI (nt, nt viruses, nt prokaryotes, nt eukaryotes, nt others) using MegaBLAST. These entries were used as reference sequences in the RefSeq database for NCBI and therefore should have aligned with sequences from the same species or strain. These bacterial sequences did not align to sample sequences of the same or similar species, only to enterovirus species. 30 entries and sequence blastn results are shown in the PASS/DPS repository https://github.com/CDCgov/PASS/tree/master/DPS
